# Characterizing the Antimicrobial Function of a Dairy-Originated Probiotic, *Propionibacterium freudenreichii*, Against Multidrug-Resistant *Salmonella enterica* Serovar Heidelberg in Turkey Poults

**DOI:** 10.3389/fmicb.2018.01475

**Published:** 2018-07-12

**Authors:** Divek V. T. Nair, Anup Kollanoor Johny

**Affiliations:** Department of Animal Science, University of Minnesota, Saint Paul, MN, United States

**Keywords:** probiotic, turkey safety, antibacterial, antibiotic alternative, *Propionibacterium*, *Salmonella* Heidelberg, multidrug-resistant

## Abstract

Antimicrobial potential of a dairy-origin probiotic bacteria, *Propionibacterium freudenreichii*, against multidrug-resistant *Salmonella* Heidelberg (SH) in turkey poults was determined in the current study. Employing *in vitro* experiments, two strains (subsp.) of *P. freudenreichii*: *P. freudenreichii freudenreichii* B3523 (PF) and *P. freudenreichii shermanii* B4327 (PS) were tested for their ability to resist low pH (2.5) and bile salts (0.3%). In addition, the ability of the strains to adhere to and invade avian epithelial cells was determined after exposure to *Propionibacterium* strains followed by SH challenge. Moreover, the antibacterial activity of the strains’ cell-free culture supernatants (CFCSs) were tested against three major foodborne pathogens, including SH. Furthermore, the susceptibility of the strains to common antibiotics used for human therapy was determined. The hemolytic properties of the strains were determined in comparison to *Streptococcus pyogenes*, a known hemolysis-causing pathogen. Appropriate controls were kept in all studies. Using two *in vivo* experiments, PF was tested against SH colonization of poult ceca and dissemination to liver and spleen. The four treatment groups were: negative control, PF control (PFC), SH control (SC), and a test group (PFS; PF + SH). The poults in the PFC and PFS groups were inoculated with 10^10^ CFU ml^−1^ PF on day 1 through crop gavage and subsequently supplemented through drinking water. On day 7, SC and PFS groups were challenged with SH at 10^6^ CFU ml^−1^, and after 7 days, cecum, liver, and spleen were collected for determining surviving SH populations. Results indicated that both PF and PS resisted pH = 2.5 and 0.3% bile salts with surviving populations comparable to the control and adhered well onto the avian epithelial cell lines. The strains were susceptible to antibiotics and did not invade the epithelial cells or exhibit hemolytic properties. The CFCSs were highly bactericidal against all tested pathogens. In turkey poults, PF significantly reduced cecal colonization of SH and the dissemination of the pathogen to the liver, compared to the SH challenge controls (*P* < 0.05). Results revealed that PF, a non-host gastrointestinal tract-derived probiotic, could be an antibiotic alternative to prevent the early colonization of SH in poults, improving the preharvest safety of turkeys.

## Introduction

Non-typhoidal *Salmonella* is a major bacterial pathogen that causes ∼11% illnesses, 35% hospitalizations, and 28% deaths associated with foodborne outbreaks in the United States, annually (Scallan et al., 2011). Poultry serve as natural reservoir hosts for *Salmonella* and poultry products are commonly implicated in related outbreaks (CDC, 2013; Andino and Hanning, 2015; Antunes et al., 2016). *Salmonella* can survive and colonize in the gastrointestinal tract (GIT) of poultry. Once colonized, *Salmonella* could be shed through their feces leading to environmental (farm) contamination, transmission of the pathogen to fresh incoming flocks, or cross-contamination of the carcasses during faulty evisceration (Foley et al., 2011; Antunes et al., 2016).

*Salmonella* Heidelberg (SH) is an emerging *Salmonella* serovar in poultry, including turkeys, that has high colonization potential and invasion ability (Nair et al., 2018) compared to the most prevalent serovars such as *S.* Enteritidis (Borsoi et al., 2011). Multistate outbreaks of foodborne salmonellosis occurred in 2011, 2013, and 2014 due to the consumption of poultry products, including ground turkey contaminated with multidrug-resistant (MDR) SH (CDC, 2011, 2014a,b). Moreover, the occurrence of MDR SH in turkeys is a serious concern since it is a major poultry species produced and consumed in the United States and exported globally in massive volumes (USPEA, 2017). This situation is aggravated due to the increased invasiveness of SH isolated from poultry and poultry products, and their multiple drug resistance profiles (Foley et al., 2011; Medeiros et al., 2011; Hoffmann et al., 2014; Antunes et al., 2016). In response to the antibiotic resistance development in foodborne bacteria isolated from animal production, the United States Food and Drug Administration (USFDA) has introduced the Veterinary Feed Directive (VFD) that necessitates veterinary supervision for therapeutic and metaphylactic use of antibiotics in food animals, including poultry (FDA, 2015). This step has resulted in the rigorous search for antibiotic alternatives that can produce meaningful reductions of pathogenic bacteria in food animals, thereby reducing the risk of contaminated animal products entering the food chain.

Dairy-origin *Propionibacterium* are primarily isolated from milk and milk products and ruminants, including dairy cattle (Gutierrez, 1953; Rossi and Dellaglio, 2007; Quigley et al., 2013). These Gram-positive, non-motile bacteria have been used as probiotics in humans with long-term sustainable activity and ability to produce short-chain fatty acids and other metabolites in the GIT (Huang and Adams, 2004). More importantly, *Propionibacterium freudenreichii* spp. is well-characterized among the dairy *Propionibacterium* for its widespread application in the food industry, including production of vitamins, ripening of cheese, and as probiotics (Falentin et al., 2010; Thierry et al., 2011; Cousin et al., 2012; Zárate, 2012; Argañaraz-Martínez et al., 2013; Ganan et al., 2013; Yuksekdag et al., 2014; Rabah et al., 2017). They are classified as a Generally Recognized as Safe (GRAS) and Qualified Presumption of Safety (QPS) status bacteria for use in foods (EFSA, 2013; FDA, 2014). Recently, we found that that *P. freudenreichii* subsp. *freudenreichii* (PF) and *P. freudenreichii* subsp. *shermanii* (PS) have anti-virulence property against *Salmonella* spp., including MDR SH *in vitro* (Nair and Kollanoor-Johny, 2017a). So far, no studies have been conducted to determine the efficacy of *P. freudenreichii* against MDR SH in poultry, including turkeys.

A successful probiotic bacterium should traverse through the adverse digestive and absorptive environment of the poultry GIT to render its beneficial effect on the host. In this process, it should withstand several stresses, including low pH and bile resistance in the intestinal tract (Riley and Austic, 1984; Mahagna et al., 1995; Ao, 2005; Ao et al., 2008; Svihus, 2014). In addition, the probiotic bacterium should possess high affinity and adherence to intestinal epithelial cells and produce secondary metabolites that are responsible also for the antibacterial activity (Patterson and Burkholder, 2003; Alloui et al., 2013; Surendran Nair et al., 2017). Furthermore, the probiotic organism should not develop resistance to commonly used antibiotics and develop pathogenicity in the host species (Salminen et al., 1998; Lin et al., 2007; Musikasang et al., 2009; Smith, 2014; Harimurti and Hadisaputro, 2015). Given these factors, the selection of a potential probiotic is dependant on its tolerance to host physiological stresses since the probiotic qualities are highly strain dependent (Klaenhammer and Kullen, 1999; Saarela et al., 2000; Tuomola et al., 2001; Dunne et al., 2001; Rinkinen et al., 2003; Zárate, 2012). Therefore, the functional properties of the candidate strains of *P. freudenreichii* spp., especially those characteristics that aid in exhibiting antimicrobial activity in the poultry GIT, need to be evaluated to ensure safe application in poultry, in our case, turkeys.

The objectives of the current study, therefore, were (1) to determine the ability of dairy-origin PF and PS to resist various GIT stressors for effective colonization and exhibit antimicrobial activity, *in vitro* and (2) to validate the antimicrobial efficacy of PF on MDR SH colonization of the cecum, and the dissemination of the pathogen to the liver and spleen of turkey poults.

## Materials and Methods

### Ethics Statement

The poult experiments were approved by the Institutional Animal Care and Use Committee, and the use of infectious agents in the experiments was approved by the Institutional Biosafety Committee at the University of Minnesota.

### Bacterial Strains and Culture Conditions

#### Propionibacterium freudenreichii

Two strains of *P. freudenreichii* were used in the study: *P*. *freudenreichii* subsp. *freudenreichii* B3523 (hereafter PF; USDA ARS NRRL Culture Collection, Peoria, IL, United States) and *P. freudenreichii* subsp. *shermanii* B4327 (hereafter PS; USDA ARS NRRL Culture Collection, Peoria, IL, United States). One hundred microliter of PF or PS stock culture was grown in 10 ml of de Man–Rogosa–Sharpe broth (MRS; catalog no. C5932, Criterion, Hardy Diagnostics, Santa Maria, CA, United States) for 18 h at 41°C. The culture was washed twice with 10 ml of phosphate buffered saline (PBS, pH 7.2) and sedimented by centrifugation (3,600 × *g*, 4°C, 15 min; Allegra X-14R, Beckman Coulter, South Kraemer Boulevard, CA, United States). The pellet was resuspended in 10 ml of PBS, and the bacterial populations in the culture were confirmed by plating 0.1 ml of appropriate dilutions on MRS plates. Viable PF and PS populations were determined after incubating the plates at 41°C (turkey body temperature) for 48 h (Nair and Kollanoor-Johny, 2017a).

Since PF and PS responded similarly in the *in vitro* experiments, PF was selected for the *in vivo* study. PF was made resistant to 50 μg ml^−1^ rifampicin (Rf; catalog no. 50-213-645, Research Products International Corp, 410 E Business Center Dr., Mt Prospect, IL 60056, United States) for selective enumeration and to avoid any confounding inherent *Propionibacterium* in the turkey GIT. The strain was confirmed for resistance to Rf by streaking on MRS containing 50 μg ml^−1^ of Rf (MRS-Rf). For determining the bacterial count, Rf-resistant strain was grown overnight aerobically in 10 ml of MRS supplemented with 50μg ml^−1^ Rf at 37°C. For inoculating birds, a 24 h, Rf-resistant PF (approximately 10^9^ CFU ml^−1^) culture was grown in 1 L MRS broth containing 50 μgml^−1^ Rf and were resuspended in 100 ml PBS after centrifugation at 15,000 rpm for 15 min at 4°C. From this, 1 ml Rf-resistant PF (approximately 10^10^ CFU ml^−1^) was used to inoculate the day-old poults using crop gavage method. On subsequent days, 10^10^ CFU ml^−1^ Rf-resistant PF was supplemented per gallon of drinking water continuously for 14 days.

#### *Salmonella* Heidelberg

A US poultry outbreak isolate of MDR SH was used in the study (GT2011; Nair and Kollanoor-Johny, 2017a,b; Nair et al., 2018). Glycerol stocks of SH stored at −80°C were used for the preparation of working cultures. From the stock cultures, 100 μl was inoculated to 10 ml tryptic soy broth (TSB; catalog no. C7141, Criterion, Hardy Diagnostics, Santa Maria, CA, United States) and incubated for 24 h at 37°C. After three sub-cultures, the third-generation cultures were washed with PBS, centrifuged (3,600 × *g*, 15 min, 4°C) and resuspended in 10 ml PBS. Then the bacterial culture in PBS was serially diluted (1:10) to get a final concentration of 10^7^ CFU ml^−1^. From this, 100 μl was used in the experiments to inoculate the wells containing 2 ml TSB (Kollanoor Johny et al., 2010; Nair and Kollanoor-Johny, 2017a,b; Nair et al., 2018). For the *in vivo* study, GT2011 was made resistant to 50 μg ml^−1^ nalidixic acid sodium salt (NA; CAS. no. 3374-05-8, Alfa Aesar, Haverhill, MA, United States) for selective enumeration to avoid any confounding inherent SH in the turkey GIT. In addition, since the resistance is plasmid encoded for the 2011 ground turkey outbreak strains, any confounding due to the potential loss of plasmids in the GIT was taken care by making the strain NA-resistant. The NA-resistant strain (GT2011NAL; Nair and Kollanoor-Johny, 2017b; Nair et al., 2018) were confirmed for resistance to NA by streaking on xylose lysine desoxycholate (XLD; catalog no. C7322, Criterion, Hardy Diagnostics, Santa Maria, CA, United States) containing 50 μg ml^−1^ of NA (XLD-NA). For inoculating poults, GT2011NAL was grown in 100 ml TSB, and a 16 h broth culture (approximately 10^9^ CFU ml^−1^) was centrifuged (3,600 × *g*, 15 min, 4°C), and the pellet was resuspended in sterile 100 ml PBS (pH 7.2). The culture was serially diluted in PBS to reach final concentration of 10^6^ CFU ml^−1^. Then 2 ml of 10^6^ CFU ml^−1^ GT2011NAL was used to inoculate the poults using crop gavage method (Kollanoor-Johny et al., 2012a; Nair et al., 2018).

#### *Escherichia coli* O157: H7

*Escherichia coli* O157: H7 strain CDC EDL 933 (ATCC 43895, Manassas, VA, United States) was used in the study. From the glycerol stock, 100 μl was inoculated to 10 ml TSB and incubated for 24 h at 37°C. After sub-culturing, the third-generation cultures were washed with PBS, centrifuged (3,600 × *g*, 15 min, 4°C) and resuspended in 10 ml PBS. Then the bacterial culture in PBS was serially diluted (1:10) to get a final concentration of 10^7^ CFU ml^−1^. From this, 100 μl was used in the experiments to inoculate the wells containing 2 ml TSB (Amalaradjou et al., 2010; Surendran Nair et al., 2016a,b).

#### Listeria monocytogenes

*Listeria monocytogenes* serotype 4b (ATCC) was used in the study. A volume of 100 μl *monocytogenes* inoculum from the glycerol stock cultures were transferred to 10 ml TSB and incubated for 24 h at 37°C to prepare working cultures. After sub-culturing, the third-generation cultures were washed with PBS, centrifuged (3,600 × *g*, 15 min, 4°C), and resuspended in 10 ml PBS. Then the bacterial culture in PBS was serially diluted (1:10) to get a final concentration of 10^7^ CFU ml^−1^. From this, 100 μl was used in the experiments to inoculate the wells containing 2 ml TSB (Upadhyay et al., 2015; Nair and Kollanoor-Johny, 2017a).

### *In Vitro* Study

#### Determination of Probiotic Resistance to Low pH

Strain PF or PS was grown separately in MRS broth for 18 h at 41°C. The bacterial culture was washed twice with PBS after centrifugation at 3,600 × *g* and 4°C for 15 min. Then the bacterial pellet was resuspended in 10 ml PBS with a pH adjusted to 2.5 using 0.1 N HCl. Bacterial pellet resuspended in PBS with a pH of 7.2 served as negative control. The control and treatments were incubated at 41°C for 3.5 h. Then at 0 and 3.5 h of incubation, the samples were serially diluted, and 100 μl of appropriate dilutions were plated on MRS agar plates. The survival of PF and PS was determined by enumerating the viable bacteria on plates after 48 h of incubation at 41°C, separately (Owusu-Kwarteng et al., 2015). Duplicate samples were included for each treatment, and the experiment was repeated at least three times.

#### Determination of Probiotic Resistance to Bile Salts

Strain PF or PS was grown separately in MRS broth for 18 h at 41°C. The bacterial culture was washed twice with PBS after centrifugation at 3,600 × *g* and 4°C for 15 min. Then the bacterial pellets were resuspended in 10 ml PBS containing 0.3% bile salt and an adjusted pH of 8.0 using 0.1 N NaOH. Bacterial pellet resuspended in PBS (pH = 7.2) without bile salt served as negative control. The control and treatments were incubated at 41°C for 3.5 h. Then at 0 and 3.5 h of incubation, the samples were serially diluted, and 100 μl of appropriate dilutions were plated on MRS plates. The survival of PF and PS was determined by enumerating viable bacteria on the plates after 48 h of incubation at 41°C, separately (Owusu-Kwarteng et al., 2015). Duplicate samples were included for each treatment, and the experiment was repeated at least three times.

#### Determination of Probiotic Hemolytic Activity

Strain PF or PS was grown separately in MRS broth for 18 h at 41°C. The bacterial culture was washed twice with PBS after centrifugation at 3,600 × *g* and 4°C for 15 min. Then the cultures were streaked on Columbia blood agar [Columbia agar (Criterion, Hardy Diagnostics, CA, United States) + 5% (w/v) defibrinated turkey blood (Rockland Immunologicals, PA, United States)]. Columbia blood agar streaked with *Streptococcus pyogenes* (Hardy Diagnostics) with known hemolytic activity was kept as positive control whereas Columbia blood agar streaked with PBS served as negative control (Owusu-Kwarteng et al., 2015). Duplicate samples were included for each treatment, and the experiment was repeated at least three times.

#### Determination of Probiotic Antimicrobial Activity

Strain PF or PS was grown in MRS broth separately for 72 h at 41°C. The cultures were filter sterilized through a 0.22 μm filtration apparatus to prepare cell-free culture supernatant (CFCS) of PF or PS. The antimicrobial activity of CFCS was determined against three major foodborne pathogens MDR SH, *L. monocytogenes* and *E. coli* O157: H7. The experiment was conducted using 24 well tissue culture plates. A 100 μl bacterial culture at 10^7^ CFU ml^−1^ (SH, *L. monocytogenes*, or *E. coli* O157: H7) was added to the wells containing 2 ml TSB having either 5, 10, 15, or 20% (100, 200, 300, and 400 μl; v/v) of CFCS in a 24-well culture plate. Since a lowering in pH was observed in TSB while adding CFCS, the controls were adjusted with 0.1 N HCl to match the pH of treatments containing CFCS and inoculated with the pathogens. Then the treatments and pH-adjusted controls were incubated at 41°C for 24 h, and the optical density (OD = 600 nm) reading was taken at 0 and 24 h (Owusu-Kwarteng et al., 2015; Nair and Kollanoor-Johny, 2017a). Duplicate samples were included for each treatment, and the experiment was repeated at least three times.

#### Determination of Probiotic Susceptibility to Antibiotics

The antibiotic susceptibility of PF and PS was tested against the common antibiotics that have interpretative criteria either with European Food Safety Authority (EFSA) or Clinical and Laboratory Standards Institute (CLSI) for evaluating minimum inhibitory concentration (MIC; CLSI, 2005, 2015) breakpoints or microbiological cut-off values (MCV; EFSA, 2012). Interpretative MIC or MCV criteria were first sought for *Propionibacterium*, followed by *Lactobacillus* for meaningful comparisons. If criteria were not available for both, interpretation was not expanded comparing *Streptococcus* Groups A, B, C, and G, or other Gram-positive anaerobes. The interpreted antibiotics in the current study include Penicillin (Class – Penicillins; tested range – 0.06–8 μg mL^−1^; MIC ≤ 8 μg mL^−1^ susceptible; for *Lactobacillus*, no criteria for *Propionibacterium*), Amoxicillin (Class – Penicillins; tested range – 0.25–16 μg mL^−1^; MIC ≤ 2 μg mL^−1^ susceptible to Ampicillin; same interpretative criteria applied for MIC of Amoxicillin for *Lactobacillus* as per CLSI, 2015), Clindamycin (Class – Lincosamides; tested range – 0.5–4 μg mL^−1^; MCV = 0.25 μg mL^−1^ susceptible), Erythromycin (Class – Macrolides; tested range – 0.12–4 μg mL^−1^; MCV = 0.5 μg mL^−1^ susceptible), Gentamicin (Class – Aminoglycosides; tested range – 0.5–8 μg mL^−1^; MCV = 64 susceptible), Streptomycin (Class – Aminoglycosides; tested range – 8–1024 μg mL^−1^; MCV = 64 μg mL^−1^ susceptible; **Table 1**, EFSA, 2008), and Tetracycline (Class – Tetracyclines; tested range – 0.25–8 μg mL^−1^; MCV = 2 μg mL^−1^ susceptible). The tests were conducted using Sensititre^TM^ plates (Trek Diagnostic Systems, Thermo Fisher Scientific, Waltham, MA, United States).

**Table 1:** Susceptibility of *P*. *freudenreichii* (PF) and *P. shermanii* (PS) to antibiotics that have MCV or MIC interpretative criteria as per EFSA (2013) or CLSI (2005, 2015), respectively.

Antibiotics	PF	PS
	(MIC-μg ml^−1^)	(MIC-μg ml^−1^)
*Penicillins*		
Amoxicillin	0.50	1.00
Penicillin	0.25	0.50
*Lincosamides*		
Clindamycin	<0.50	<0.50
*Macrolides*		
Erythromycin	<0.12	<0.12
*Aminoglycosides*		
Gentamicin	<0.50	<0.50
Streptomycin	<8.00	<8.00
*Tetracyclines*		
Tetracycline	<0.25	<0.25

### Determination of Potential of Probiotics to Adhere (Associate) to Epithelial Cells

#### Cell-Association Assay

The adhesion of PF and PS to avian epithelial cells was determined using Budgerigar Abdominal Tumor Cells (BATCs). The bacterial strains were grown separately in sterile cecal filtrate and continuously sub-cultured for three generations at 41°C in 5% CO_2_ with agitation (100 rpm) to reach a concentration of 10^9^ CFU ml^−1^. After overnight incubation, cecal filtrate containing 10^9^ CFU ml^−1^ PF or PS was used as the inoculum. Sterile turkey cecal filtrates were used for cell association experiments to mimic the cecal environment. The cecal filtrate with PF or PS was added to the wells containing BATCs (10^5^ cells/well, >95% confluence; Kollanoor-Johny et al., 2012a; Nair and Kollanoor-Johny, 2017a) in Dulbecco’s modified Eagle medium (DMEM, Invitrogen, Carlsbad, CA, United States) and incubated at 37°C under 5% CO_2_ for 2 h. Then the wells were washed with DMEM three times, added with 0.1% Triton-X (Invitrogen, Carlsbad, CA, United States) and incubated at 37°C for 15 min. The Triton-X treated cells were homogenized, and cell homogenates were plated on MRS agar plates. The cell-adhered PF or PS was determined after incubating the plates at 37°C for 24 h (Nair and Kollanoor-Johny, 2017a). Duplicate samples were included for each treatment, and the experiment was repeated at least three times.

#### Gentamicin Protection (Epithelial Invasion) Assay

The BATCs were also used to study the invasion potential of PF and PS to BATCs (Nair and Kollanoor-Johny, 2017a). Briefly, the BATCs were pre-exposed to 10^9^ CFU ml^−1^ of PF or PS, separately, for 2 h at 37°C under 5% CO_2_ in DMEM: sterile cecal filtrate (1:1). Then the wells were washed three times with DMEM, and fresh whole medium containing 100 μg mL^−1^ of gentamicin (Catalog no. 15750078; Gibco, Invitrogen, Carlsbad, CA, United States) was added to the wells. The wells were incubated at 37°C for 1 h to kill cell surface-attached bacteria. Then the wells were washed with PBS and treated with 0.1% Triton-X to lyse the BATC. After incubation at 37°C with 5% CO_2_ for 15 min, the cell homogenates were plated on MRS. The invasion of PF and PS was determined by enumerating viable bacteria after incubating the plates at 37°C for 24 h. Duplicate samples were included for each treatment, and the experiment was repeated at least three times.

### *In Vivo* Study

#### Experimental Birds, Housing, and Experimental Design

Day-of-hatch, commercial turkey poults (Hybrid Converter), male and female in equal, purchased from a commercial hatchery in Minnesota, were weighed and allocated to isolators in the Research Animal Resources biocontainment (isolation) units at the University of Minnesota. The isolators were maintained with adequate light, heat and floor space specific to the age group. The birds were supplied with *Salmonella-*free *ad libitum* feed (Famo Feeds Inc., 446 Industrial Dr., Freeport, MN, United States) and water according to NRC recommendations.

Two experiments were conducted. In each experiment, day-old poults (*N* = 48) were randomly distributed in four isolator pens with 12 birds each. The treatment groups were: negative control (NC; poults without PF supplementation or SH challenge), PF control (PFC; poults with PF supplementation and without SH challenge), SH control (SC; poults challenged with SH and without PF supplementation), and test group (PFS; poults supplemented with PF and challenged with SH). On day 1, the fecal droppings of the poults from each group were examined for inherent SH, if any. On day 1, the poults in the PFC and PFS groups were inoculated with 10^10^ CFU ml^−1^ PF using crop gavage method. On subsequent days, these groups were supplemented with PF through drinking water. On day 7, SC and PFS groups were challenged with SH at 10^6^ CFU ml^−1^ by crop gavage (Nair et al., 2018). Two days after challenge, two poults from each group were euthanized to ensure cecal colonization of SH. Cecum, liver, and spleen were collected to determine PF and SH colonization in the cecum, and SH dissemination to the liver and spleen. The remaining poults from all four groups were euthanized on day 14, and cecum, liver, and spleen were collected for microbiological analysis.

#### Determination of PF and SH in Turkey Poult Cecum

The colonization of PF and SH in poults were determined after collecting the cecum in 10 ml PBS. The samples were homogenized, serially (1:10) diluted in PBS and 200 μl from appropriate dilutions were plated on MRS-Rf and XLD-NA agar for PF and SH, respectively. Additionally, all samples were enriched in 10 ml selenite cysteine broth (SCB, Hardy Diagnostics, Santa Maria, CA, United States), aerobically, and incubated at 37°C for 6 h, and streaked on XLD-NA plates. The XLD-NA plates were then aerobically incubated at 37°C for 24 h (Kollanoor Johny et al., 2009, 2010; Kollanoor-Johny et al., 2012a,b; Nair and Kollanoor-Johny, 2017a,b).

#### Determination of SH in Liver and Spleen of Turkey Poults

The liver and spleen samples collected on day 14 were enriched in 10 ml SCB. Enriched samples were incubated 37°C. After 8–12 h incubation, the enriched samples were streaked on XLD and XLD-NA plates, and incubated for 24 h at 37°C to determine the presence of SH in liver and spleen (Nair and Kollanoor-Johny, 2017b).

### Statistical Analysis

All *in vitro* experiments were repeated at least three times with duplicate samples per experimental group (*n* = 6/experiment). Each tube was considered an experimental unit for the bile salt resistance, and resistance to low pH experiments. Each well (on a 24-well culture plate) was considered as an experimental unit in the antimicrobial activity determination, and epithelial cell association and invasion assays. Differences between two independent treatments were analyzed using two-tailed *t* tests, and a *P <*0.05 was considered statistically significant. The results are provided as mean values and standard errors of the means (SEM). The *in vivo* experiment was done two times. A completely randomized design with a factorial treatment structure of 2 × 4 × 3 was used for the *in vivo* experiments. The factors included were two experiments, four treatment groups (NC, PFC, SC, and PFS), and three tissue samples (cecum, liver, and spleen). Among these, PFC and PFS groups were compared to determine the efficacy of PF colonization in the cecum. Similarly, SC and PFS were used to determine the efficacy of PF against SH colonization in the cecum. Bacterial counts were logarithmically transformed (log_10_ CFU g^−1^) before analysis. Since there were no significant difference between the experiments, liver and spleen counts data in different groups from two *in vivo* experiments were combined for analysis. On the other hand, there were significant differences between the experiments for the cecal SH counts; data from independent studies were analyzed separately. The data were analyzed using the PROC-MIXED procedure of the statistical analysis software (SAS, version 9.4, SAS Institute Inc., Cary, NC, United States). Differences among the least squares means were detected using Fisher’s least significance difference test. The liver and spleen data were analyzed using the Wilcoxon Rank Sum test in SAS to determine the effect of PF on the presence (positive after either direct plating or enrichment) or absence (negative after both direct plating and enrichment) of SH in different organ samples. A *P* < 0.05 was considered statistically significant.

## Results

### *In Vitro* Study

**FIGURE 1:**
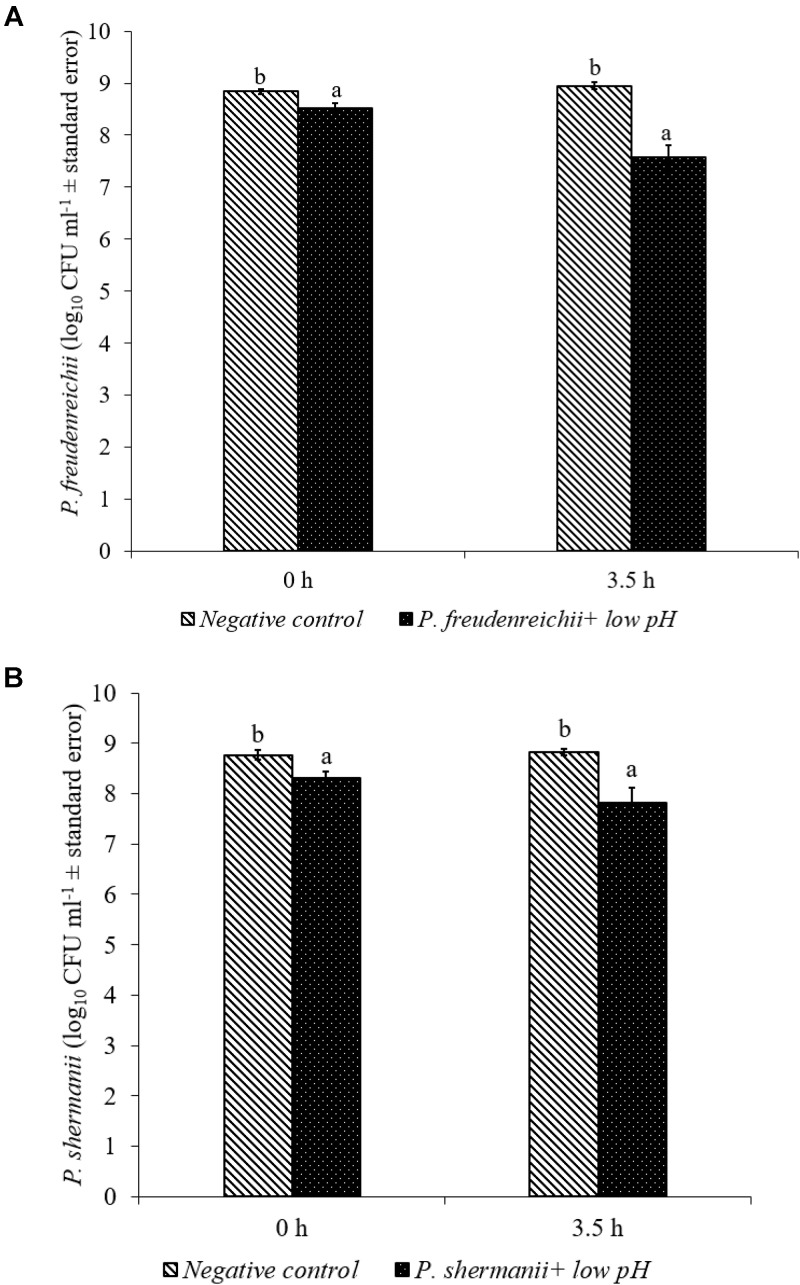
Effect of low pH on the survival of **(A)** *P*. *freudenreichii* (PF) and **(B)** *P. shermanii* (PS). *Propionibacterium* (10^9^ CFU ml^−1^) was exposed to a low pH = 2.5. Survival of *Propionibacterium* was determined after 3.5 h incubation at 41°C. *Propionibacterium* exposed to a pH = 7.2 for 3.5 h at 41°C served as negative control (*n* = 6; ^a-b^*P* < 0.05). *Propionibacterium* counts were represented as log_10_ CFU ml^−1^ ± standard error.

**FIGURE 2:**
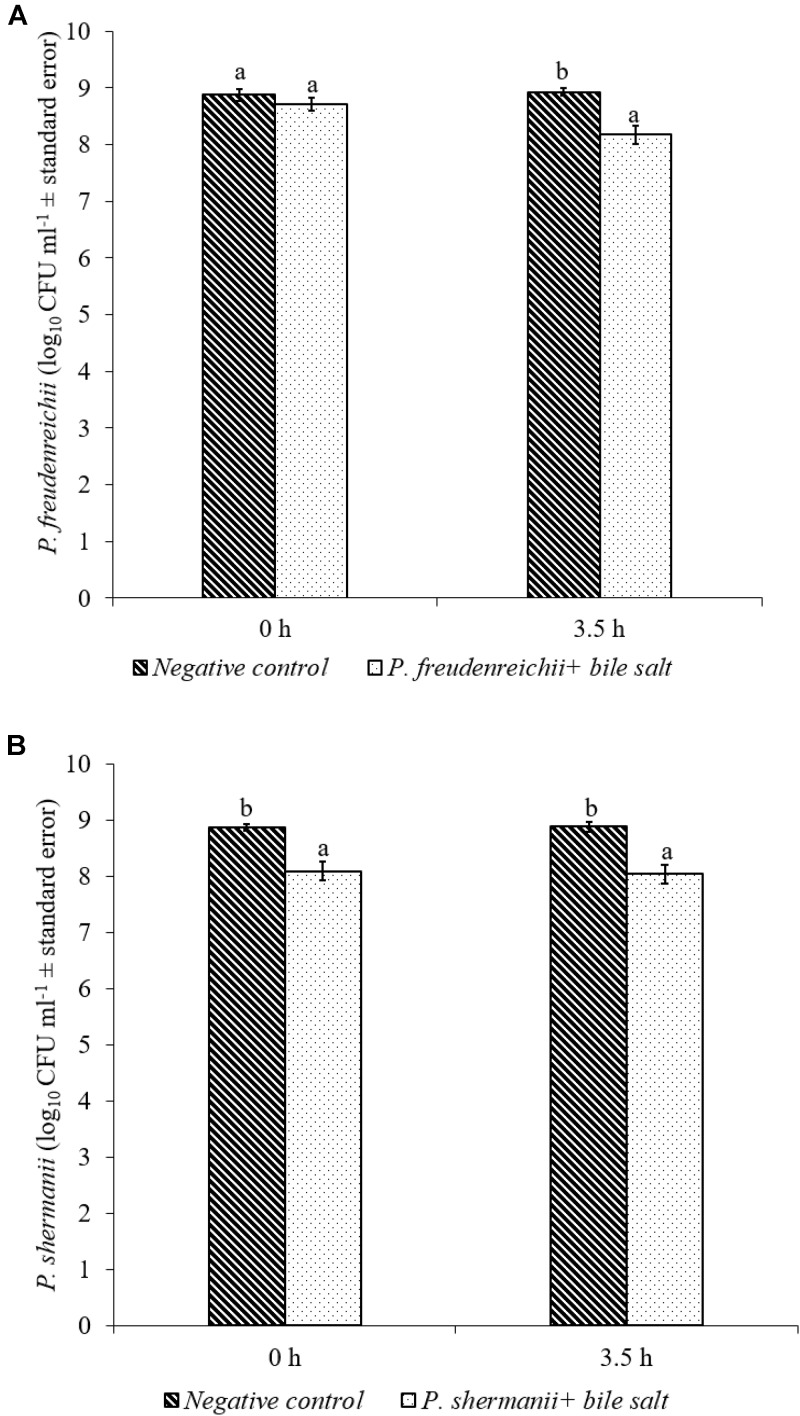
Effect of bile salt on the survival of **(A)** *P*. *freudenreichii* (PF) and **(B)** *P. shermanii* (PS). *Propionibacterium* (10^9^ CFU ml^−1^) was exposed to 0.3% bile salt at a pH = 8. Survival of *Propionibacterium* was determined after 3.5 h incubation at 41°C. *Propionibacterium* exposed to a pH = 7.2 for 3.5 h at 41°C without bile salt served as negative control (*n* = 6; ^a-b^*P* < 0.05). *Propionibacterium* counts were represented as log_10_ CFU ml^−1^ ± standard error.

In the poultry GIT, the first stress a probiotic bacterium would encounter is the low pH in the proventriculus and gizzard. The results of the current study revealed that both *P. freudenreichii* strains were highly tolerant to the low pH used in the study. The viable counts of PF and PS remained as high as 7.57- and 7.81- log_10_ CFU ml^−1^, respectively, after an exposure time of 3.5 h to a pH = 2.5. Viable populations of PF and PS in the controls (pH = 7.2) were at 8.95 and 8.83 log_10_ CFU ml^−1^, respectively (**Figures 1A,B**). Similarly, the probiotics would experience the resistance of bile salt while passing through the duodenum. We found that PF and PS exhibited high survivability with viable populations of 8.17- and 8.04- log_10_ CFU ml^−1^, respectively, in the treatments when exposed to 0.3% bile salts for 3.5 h, comparable to the controls (**Figures 2A,B**).

**FIGURE 3:**
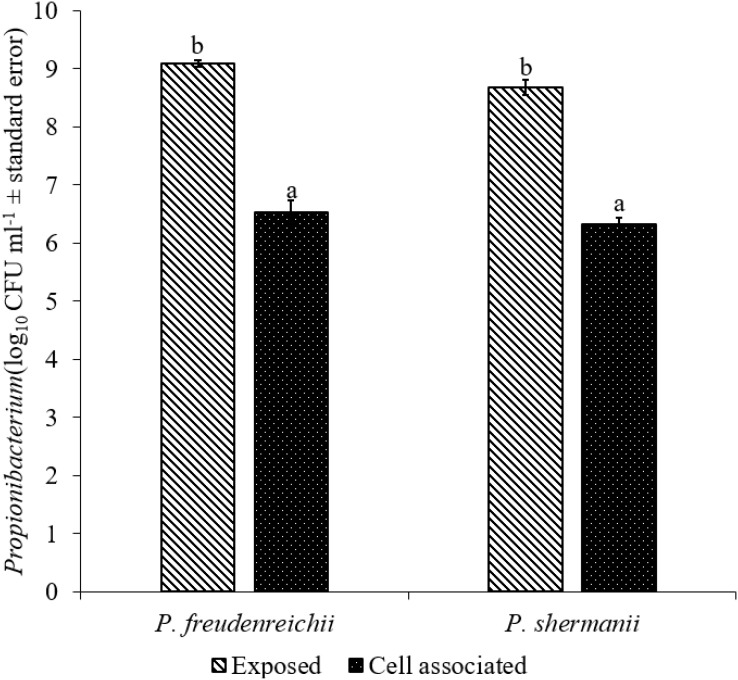
Cell association of *P*. *freudenreichii* (PF) and *P. shermanii* (PS) on BATCs. BATCs were pre-exposed to 10^9^ CFU ml^−1^ of PF or PS for 2 h at 37°C under 5% CO_2_. The adherence of the strains to BATCs (cell association) was enumerated after plating cell homogenates on MRS agar plates and incubating at 37°C for 24 h (*n* = 6; ^a-b^*P* < 0.05). *Propionibacterium* counts were represented as log_10_ CFU ml^−1^ ± standard error.

**FIGURE 4:**
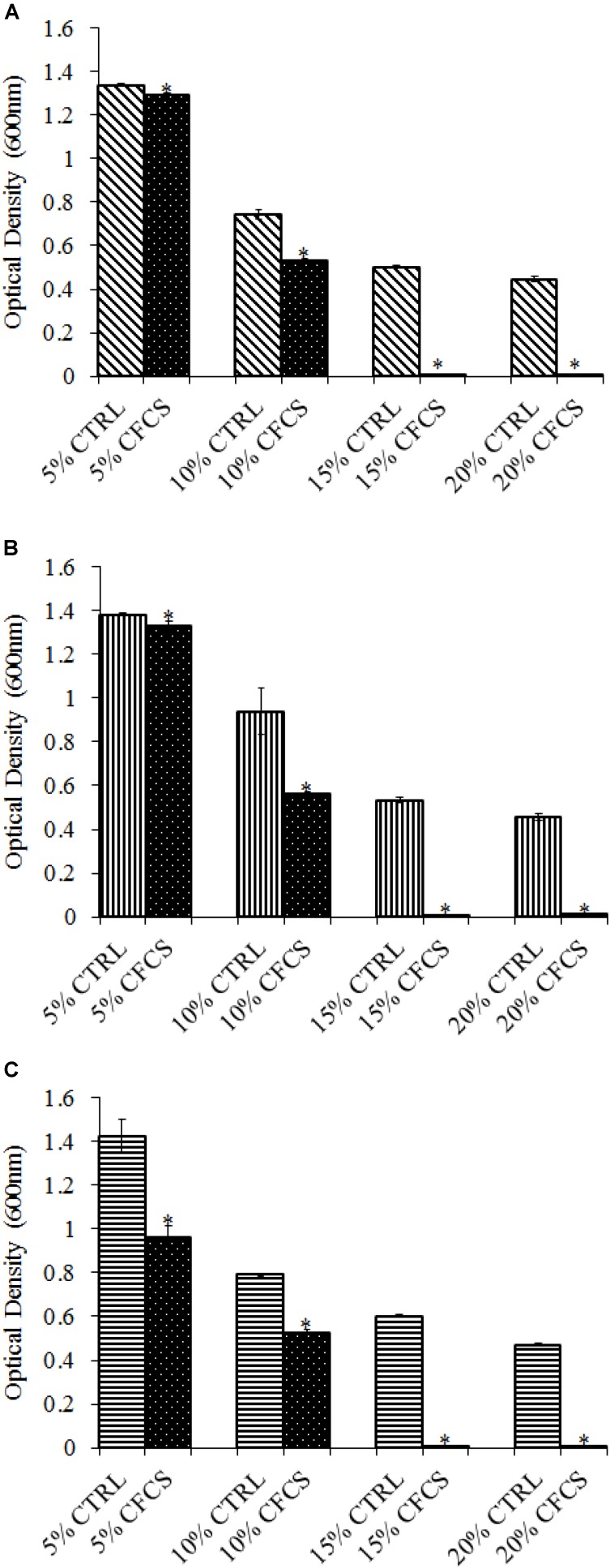
Effect of *P. freudenreichii* (PF) CFCS on **(A)** *S.* Heidelberg **(B)** *L. monocytogenes*, and **(C)** *E. coli* O157: H7. TSB containing *S.* Heidelberg, *L. monocytogenes*, or *E. coli* O157: H7 (10^6^ CFU ml^−1^) were treated with 5, 10, 15, or 20% (v/v) of cell-free culture supernatant (CFCS) of PF. The pH of TSB was adjusted to match the pH of treatments having CFCS were inoculated with the respective pathogen and used as positive controls (CTRL) for each CFCS concentration. The pathogen survival was determined by determining optical density (OD = 600 nm) after incubating samples at 41°C for 24 h (*n* = 6; ^∗^*P* < 0.05). Bacterial counts were represented as OD_600_ ± standard error.

**FIGURE 5:**
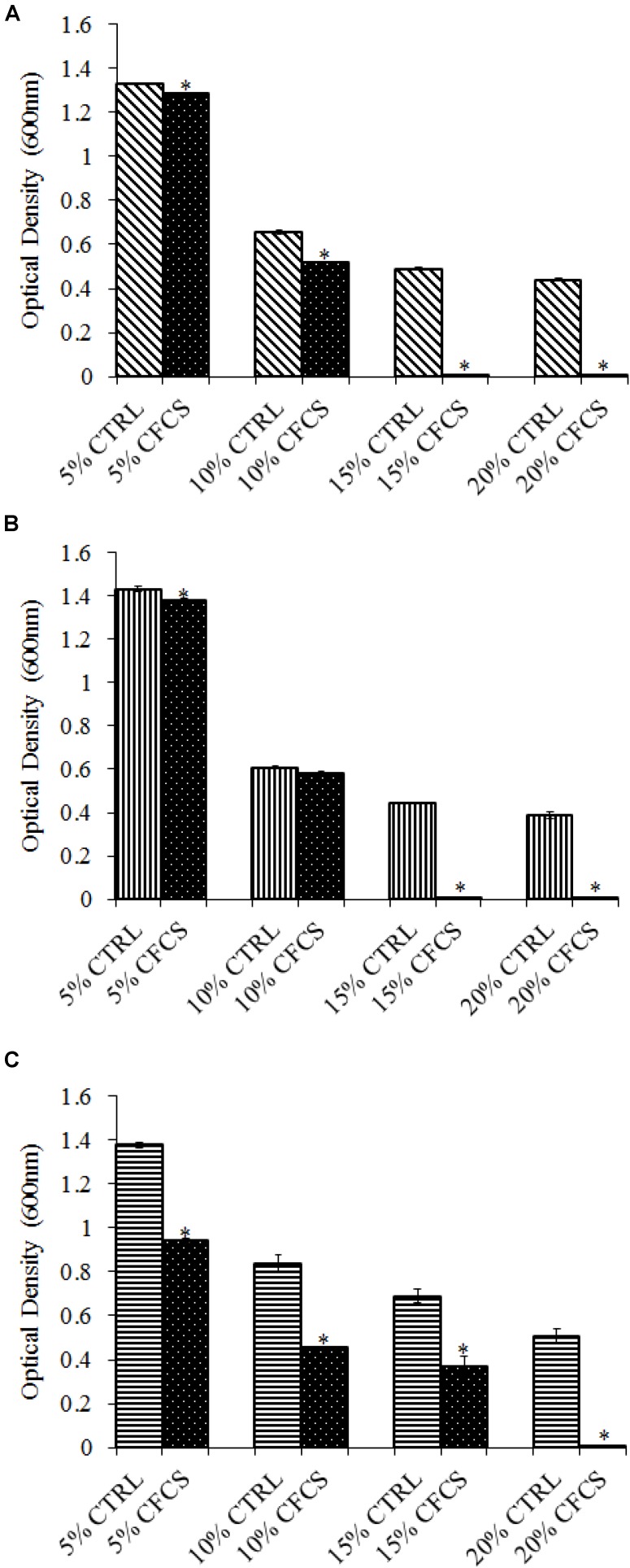
Effect of *P. shermanii* (PS) CFCS on **(A)** *S.* Heidelberg **(B)** *L. monocytogenes*, and **(C)** *E. coli* O157: H7. TSB containing *S.* Heidelberg, *L. monocytogenes*, or *E. coli* O157: H7 (10^6^ CFU ml^−1^) were treated with 5, 10, 15, or 20% (v/v) of CFCS of PS. The pH of TSB was adjusted to match the pH of treatments having CFCS were inoculated with the respective pathogen and used as positive CTRL for each CFCS concentration. The pathogen survival was determined by determining optical density (OD = 600 nm) after incubating samples at 41°C for 24 h (*n* = 6; ^∗^*P* < 0.05). Bacterial counts were represented as OD_600_ ± standard error.

The adhesion of a probiotic strain to the host GIT is necessary to elicit its prolonged effect in the host species. In the current study, both *Propionibacterium* strains showed high potential to adhere/associate to the BATCs. PF and PS adhered to BATCs at 6.5 and 6.3 log_10_ CFU ml^−1^, respectively, when exposed to ∼9.0 log_10_ CFU ml^−1^ for 2 h (**Figure 3**). Once adhered to the intestinal cells and perform colonization resistance to the invading microbes, effective probiotics would produce metabolites that may have direct bacteriostatic or bactericidal effects on the pathogens. We found that cell-free culture extracts (CFCSs) of PF and PS were effective against major foodborne pathogens, MDR SH, *E. coli* O157: H7, and *L. monocytogenes*. The CFCS at concentrations of 15 and 20% exhibited the highest antimicrobial activity followed by 10% and 5% (**Figures 4A–C**, **5A–C**).

**FIGURE 6:**
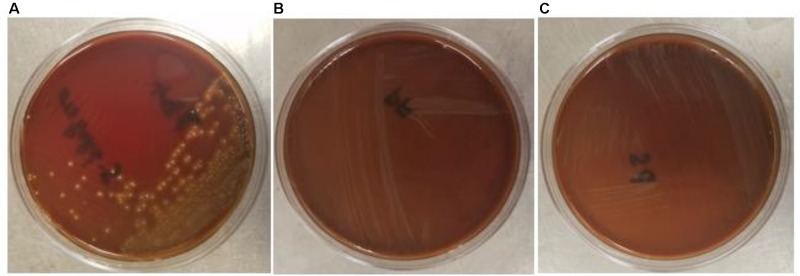
**(A–C)** Hemolytic activity of *Streptococcus pyogenes*, *P*. *freudenreichii* (PF) and *P. shermanii* (PS). PF or PS cultures (10^9^ CFU ml^−1^) were streaked on Columbia turkey blood agar. Columbia turkey blood agar streaked with *S. pyogenes* with known hemolytic activity was kept as positive control (*n* = 6; *P* < 0.05).

The safety of the probiotic strains is of utmost importance when used in animals and humans. In this regard, PF and PS did not show hemolytic activity on the Columbia blood agar (**Figures 6A–C**) confirming that the tested *P. freudenreichii* strains are safe to use in turkeys. Additionally, in our study, we observed that both strains were susceptible to the clinically important antibiotics. The minimum inhibitory concentrations (MICs) of tested *P. freudenreichii* strains were in the lower MIC range of the tested antibiotics (**Table 1**). The study also revealed that the tested *P. freudenreichii* strains were not invading the BATCs, providing a good safety margin for their use in turkeys since tissue invasion is one of the mechanisms of bacterial pathogenicity (Kollanoor-Johny et al., 2017).

### *In Vivo* Study

**FIGURE 7:**
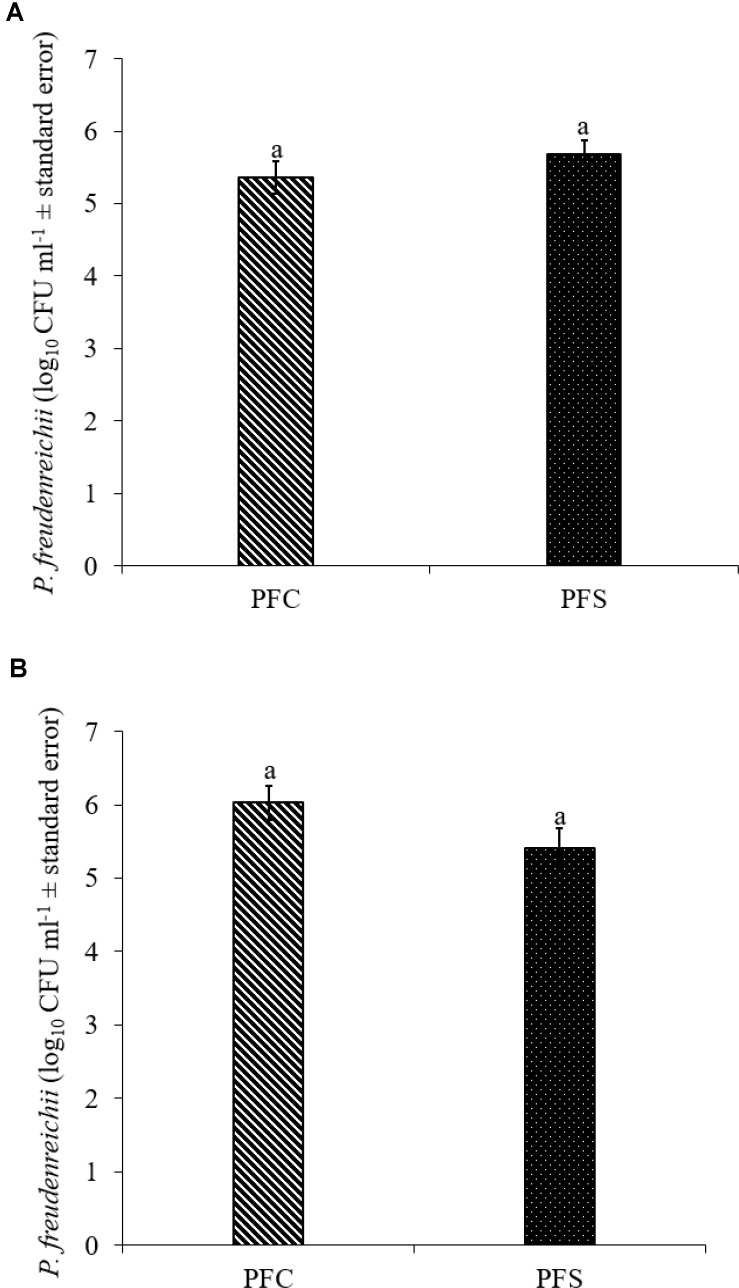
Colonization of *P. freudenreichii* (PF) in 14-day old turkey poults in experiment **(A)** 1 and **(B)** 2. The poults in PFC and PFS groups were supplemented with PF from day 1 to 14. Colonization was determined in PFC and PFS after conducting microbiological analysis of cecal samples (10 poults/group; ^a^*P*> 0.05). PFC: Poults with PF supplementation; PFS: Poults supplemented with PF and challenged with SH. PF counts were represented as log_10_ CFU ml^−1^ ± standard error.

Since PF and PS strains exhibited similar qualities *in vitro*, PF was selected for the *in vivo* experiments. In the current study, 10^10^ CFU ml^−1^ PF was supplemented per gallon of drinking water for 14 days. Of this, approximately 5.4- to 5.7- and 5.4- to 6.0- log_10_ CFU g^−1^ PF was retained in the cecum of 14-day old turkey poults that received probiotic supplementation in experiments 1 and 2, respectively (**Figures 7A,B**).

**FIGURE 8:**
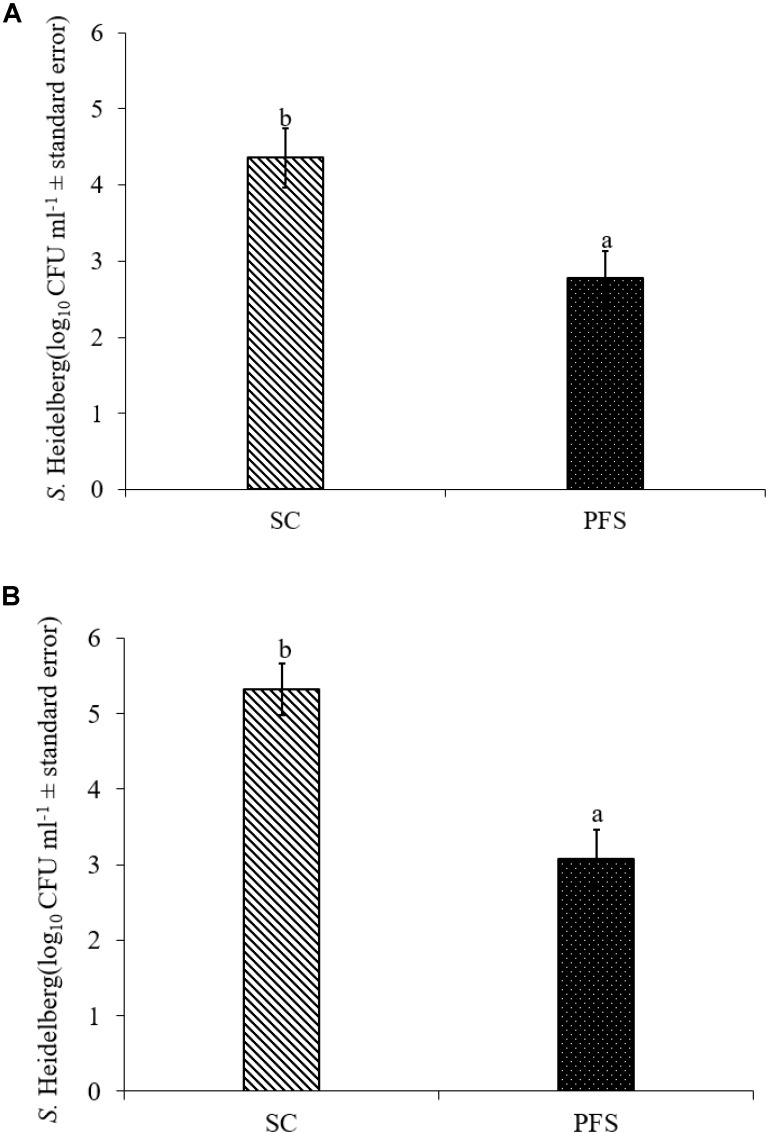
Effect of *P. freudenreichii* (PF) against MDR SH colonization on 14-day old turkey poults in experiments **(A)** 1 and **(B)** 2. The poults in SC group was challenged with SH (10^6^ CFU ml^−1^) on day 7. The PFS group was supplemented with PF from day 1 to 14 and challenged with SH on day 7. The SH recovery was determined in SC and PFS groups after conducting microbiological analysis of cecal samples (10 poults/group; ^a-b^*P* < 0.05). SC, poults challenged with SH; PFS, poults supplemented with PF and challenged with SH. SH counts were represented as log_10_ CFU ml^−1^ ± standard error.

SH colonized in high numbers in the cecum of turkey poults in the SC groups in both experiments. It was also found that PF significantly reduced SH colonization in turkey poult cecum. The supplementation of PF resulted in 1.6- and 2.2- log_10_ CFU g^−1^ reduction (*P* < 0.05) of SH on day 14 in the PFS group compared to the SC group in experiments 1 and 2, respectively (**Figures 8A,B**). Additionally, PF supplementation significantly reduced SH dissemination to the liver. On day 14, 70% liver samples were found to be positive for SH in SC groups whereas 35% SH positive liver samples were obtained in PFS groups (**Table 2**). Although, PF supplementation reduced SH dissemination to the spleen on day 14, the reductions were not significantly different (*P* = 0.069).

**Table 2:** Effect of *P. freudenreichii* (PF) against MDR SH dissemination to liver and spleen on 14-day-old turkey poults.

	SH positive samples	
Groups	Liver	Spleen
SC	14/20 (70%)	14/20 (70%)
PFS	7/20 (35%)^∗^	8/20 (40%)

*The poults in SC group was challenged with SH (10^6^ CFU ml^−1^) on day 7. The PFS group was supplemented with PF from day 1–14 and challenged with SH on day 7. The SH dissemination was determined in SC and PFS groups after conducting microbiological analysis of liver and spleen samples (10 poults/group; ^∗^indicates P < 0.05 between groups within a column).*

## Discussion

Probiotics provide benefits to a host when physiologically meaningful levels reach the GIT alive and colonize (Patterson and Burkholder, 2003; Chichlowski et al., 2007; Frese et al., 2012). Recently we found that dairy-originated PF and PS possessed the ability to reduce some of the virulence characteristics of *Salmonella* serovars in poultry, including MDR SH *in vitro* (Nair and Kollanoor-Johny, 2017a). Although much information is available on the use of *Propionibacterium* as probiotics in humans, studies that target their benefits in animals are scanty. Before testing the *Propionibacterium* strains in live poultry, we conducted a series of *in vitro* experiments to determine the qualities that might result in efficient colonization in the turkey gut and exhibit antimicrobial activity against SH.

Both *P. freudenreichii* strains were highly tolerant to low pH (**Figures 1A,B**). The underlying mechanisms of pH resistance are well studied in *P. freudenreichii* spp. The production of SCFAs could gradually reduce the pH of the medium protecting the probiotic bacteria at a pH as low as 2.0. In addition, the upregulation of biotin carboxyl carrier protein and enzymes involved in the DNA synthesis and the universal chaperonins such as GroEL and GroES could play critical roles in pH tolerance (Jan et al., 2001). Also, regulation of F_0_F_1_-ATPase enzyme, a known molecular response mechanism to low pH conditions in Gram positive organisms, has been implicated by researchers (Cotter and Hill, 2003; Fortier et al., 2003; Corcoran et al., 2005).

The *P. freudenreichii* strains used in this study showed high survival in the presence of 0.3% bile salt (**Figures 2A,B**). Adaption of *P. freudenreichii* to the stress induced by the bile has been attributed to bile salt stress-response proteins. In addition, production of superoxide dismutase and cysteine synthase were also identified in *P*. *freudenreichii* as stress response proteins mainly responding to the oxidative damage caused by bile acids (Leverrier et al., 2003). In addition, upregulation of active efflux of bile acids and salts by transporters are also implicated (Piddock, 2006; Ruiz et al., 2013).

Once the probiotic bacteria triumphs over the low pH and bile salts, the adhesion of *P. freudenreichii* spp. to intestinal cells is necessary for its colonization in the GIT and to exhibit antimicrobial effects (Patterson and Burkholder, 2003; Hossain et al., 2017). The colonization of probiotic bacteria could competitively inhibit the intestinal adhesion of pathogenic enterobacteria spp. such as *Salmonella* and *Campylobacter* (Hossain et al., 2017). In the current study, PF and PS showed higher potential to adhere to the BATCs (**Figure 3**). Once attached efficiently to the GIT, the probiotics exhibit their beneficial effects, one critical activity of importance to food safety being the antimicrobial activity against pathogenic microbes. It is reported that the cell-free supernatants derived from the probiotics are primarily responsible for the antibacterial activity due to the multitude of bioactive molecules, including bacteriocins. In line with this, the CFCSs of PF and PS were found to be active against pathogens, SH, *L. monocytogenes*, and *E. coli* O157: H7 (**Figures 4A–C**, **5A–C**). The CFCS of *P. freudenreichii* could contain SCFAs and propionicins that directly inhibit pathogens (Gwiazdowska and Trojanowska, 2006; Stackebrandt et al., 2006; Dunkley et al., 2009; Argañaraz-Martínez et al., 2013).

Safety to the host is a critical issue while considering a probiotic for human consumption or animal feeding purposes. In that regard, β-hemolytic activity is one of the characteristic features of pathogenic bacteria such as *S. pyogenes*, the reference pathogenic bacteria used in our study. The β-hemolytic activity of *S. pyogenes* is attributed to hemolysins such as streptolysin S and streptolysin O (Saroj et al., 2016; Spellerberg and Brandt, 2016). The β-hemolysis of *S. pyogenes* was evidenced by the destruction of RBCs around bacterial colonies in the blood agar plates whereas PF and PS did not show any sign of hemolysis (**Figures 6A–C**). In addition, we also evaluated the ability of *P. freudenreichii* spp. to invade the poultry epithelial cells, which is another property of pathogenic bacteria. The cell culture results indicated that *P. freudenreichii* spp. did not invade the BATCs assuring safety.

Susceptibility to common antibiotics is one of the desirable qualities of a probiotic bacterium. In our study, we observed that both strains were susceptible to the clinically important antibiotics (**Table 1**). However, the lack of extensive MIC standards for *Propionibacterium* for making meaningful interpretation of susceptibility profiles was a challenge (Mayrhofer et al., 2010). With the available MIC interpretative criteria, the tested strains were found to be susceptible to Clindamycin, Erythromycin, Gentamicin, Streptomycin, Tetracycline, and Penicillin (CLSI, 2005; EFSA, 2012). Under the molecular taxonomy model published by EFSA (2012), a probiotic with an MIC ≤cut-off could be considered acceptable as a feed additive. Since susceptibility to antibiotics depends upon the strain and/or species of probiotics (Danielsen and Wind, 2003), and lack of availability of such data in *Propionibacterium*, more studies are warranted in this area.

The *in vivo* experiments revealed that PF colonized turkey poults cecum and reduced SH colonization in the cecum (**Figures 7A,B**; *P* < 0.05), and decreased the pathogen dissemination to liver (**Table 2**, *P* < 0.05). The inhibitory action of PF on SH could be due to a competitive exclusion effect (Patterson and Burkholder, 2003; Hossain et al., 2017) or the production of secondary metabolic products, including propionate, acetate, and bacteriocins (Al-Zoreky et al., 1991; Holo et al., 2002; Argañaraz-Martínez et al., 2013) or both. Our previous study (Nair and Kollanoor-Johny, 2017a) also revealed that PF at a concentration ≥7 log_10_ CFU ml^−1^ was effective in inactivating 5 log_10_ CFU ml^−1^ SH in a co-culture medium after 24 h incubation at 37°C. Similarly, the CFCS of PF was effective in reducing the motility of SH, which is a virulence factor (Nair and Kollanoor-Johny, 2017a).

The results of the study indicated that PF reduced the dissemination of SH to liver (*P*≤ 0.05). The colonization of PF in large numbers in the cecum of turkey poults might have resulted in reduced attachment of SH to the cecum, eventually resulting in the inhibition to cross the intestinal barrier and dissemination to the internal organs. The colonization ability of PF and the resultant SH exclusion from the avian epithelial cell lines (*in vitro*) were previously proven and the current *in vivo* results corroborate with those findings (Nair and Kollanoor-Johny, 2017a). Moreover, the persistence of PF through the attachment on to the intestinal cell wall could prolong the production and release of antimicrobial metabolites such as bacteriocins (Zárate, 2012).

Overall, the results indicated that PF and PS exhibited probiotic qualities *in vitro* that could benefit their use in poultry. The tested strains showed high survival in low pH and bile salts, indicating high tolerance to the adverse GIT environment in poultry. In addition, *P. freudenreichii* spp. showed high adhesion to the avian epithelial cells. The CFCS of *P. freudenreichii* spp. exhibited antibacterial activity against major foodborne pathogens, including SH. Regarding the safety of use in turkeys, *P. freudenreichii* strains did not exhibit hemolytic properties, were susceptible to common antibiotics, and did not invade avian epithelial cells *in vitro*. Furthermore, the *in vivo* experiments revealed that PF could colonize well in the cecum of turkey poults for a period of 14 days when supplemented through drinking water that resulted in SH reduction in cecum and dissemination to liver.

## Author Contributions

AKJ conceived the idea and designed the experiments with DN. DN performed *in vitro* studies and jointly conducted the *in vivo* study with AKJ. DN conducted the statistical analysis. DN and AKJ jointly wrote the manuscript.

## Conflict of Interest Statement

The authors declare that the research was conducted in the absence of any commercial or financial relationships that could be construed as a potential conflict of interest.
